# Special Issue: “Synthesis, Characterization and Pharmaceutical Applications of Gels”

**DOI:** 10.3390/gels12020149

**Published:** 2026-02-07

**Authors:** Kalliopi Dodou

**Affiliations:** School of Health and Life Sciences, University of Teesside, Middlesborough TS1 3BX, UK; kalliopi.dodou@gmail.com

The continuous advancement and improved understanding of technologies for gel synthesis and formulation have enabled the development of novel gel systems with diverse pharmaceutical applications. This Special Issue presents a compilation of research and review papers on exciting current innovations and future trends in this field.

Bialek et al. [contribution 1] and Anaya et al. [contribution 2] explored the application of 3D printing in gel formulations. The review paper “Novel Soft Dosage Forms for Paediatric Applications: Can We 3D-Print Them or Not?” reports on the emergence of personalized pediatric gel-based formulations using 3D printing [contribution 1]. The research article “Origanum vulgare ssp. hirtum: From Plant to 3D-Printed Gummies with Antioxidant and Anti-Inflammatory Properties” shows how a culinary herb can be transformed into a healthcare product via semi-solid extrusion 3D printing [contribution 2].

Zhang et al. [contribution 3] and Boateng & Khan [contribution 4] explored the use of hydrophilic gels for wound therapy. The review paper “Multifunctional Metal Composite Hydrogels for Diabetic Wound Therapy” explores how the incorporation of metals, such as copper, silver, iron, and gold, in hydrogel scaffolds can be beneficial in the wound healing process [contribution 3]. The authors stipulate that these benefits should be balanced against potential toxicity arising from the absorption of these metals into the systemic circulation [contribution 3]. The research article “Composite HPMC-Gelatin Films Loaded with Cameroonian and Manuka Honeys Show Antibacterial and Functional Wound Dressing Properties” presents a novel honey-loaded gel film that is suitable for low-to-medium-exuding wounds. Further research is required to confirm the suitability of these gel films for treating infected chronic wounds [contribution 4].

Ke et al. [contribution 5] applied gel technology in a microneedle design for transdermal drug delivery. In their research article “Dexmedetomidine-Loaded Hydrogel Microneedles Alleviate Acute Inflammatory Visceral Pain in Mice”, they present a novel microneedle patch with analgesic efficacy as an alternative to intravenous analgesia [contribution 5].

Platelet-rich gel (PRG) supernatants of animal origin are useful bioproducts that are being piloted for the treatment of various pathological conditions such as wounds, infections, and musculoskeletal disorders, in both human and veterinary systems. The research article by Carmona & Lopez [contribution 6], “Effects of Temperature and Time on the Denaturation of Transforming Growth Factor Beta-1 and Cytokines from Bovine Platelet-Rich Gel Supernatants”, presents studies on the stability of a PRG bioproduct intended for the treatment of mastitis in dairy cows; storage of the bioproduct at room temperature prevented the denaturation of the therapeutic peptide mediators. The authors point out that this research is still at its early stages, and further research should focus on the toxicological safety of the milk produced by the treated cows for human consumption. The research paper by Ospina et al., “Short-Term Effects of Two COX-2 Selective Non-Steroidal Anti-Inflammatory Drugs on the Release of Growth Factors and Cytokines from Canine Platelet-Rich Gel Supernatants” [contribution 7], reported the interaction between a PGR bioproduct for the treatment of osteoarthritis in dogs with the NSAIDs carprofen and firocoxib. Their results prove there is a contraindication and that the administration of NSAIDs should be avoided.

Hoshi et al. [contribution 8] designed an innovative hollow gel capsule as a platform for enzymatic reactions. Their research article “Encapsulation of HRP-Immobilized Silica Particles into Hollow-Type Spherical Bacterial Cellulose Gel: A Novel Approach for Enzyme Reactions within Cellulose Gel Capsules” presents their pioneering findings, and it marks a new era for enzyme-mediated therapeutics and enzymatic drug delivery utilizing cellulose capsule gels that are shielded from immune-cell attack.

In conclusion, gel technology is being intertwined with other emerging technologies to create novel therapeutic systems. I envisage that the papers in this Special Issue will inspire and set the tone for future research in this field.

